# Microtubule-associated protein MAP1LC3C regulates lysosomal exocytosis and induces zinc reprogramming in renal cancer cells

**DOI:** 10.1016/j.jbc.2023.104663

**Published:** 2023-03-30

**Authors:** Rita Verma, Parul Aggarwal, Megan E. Bischoff, James Reigle, Dina Secic, Collin Wetzel, Katherine VandenHeuvel, Jacek Biesiada, Birgit Ehmer, Julio A. Landero Figueroa, David R. Plas, Mario Medvedovic, Jarek Meller, Maria F. Czyzyk-Krzeska

**Affiliations:** 1 Department of Cancer Biology, University of Cincinnati College of Medicine, Cincinnati, Ohio, USA; 2 Department of Biomedical Informatics, University of Cincinnati College of Medicine, Cincinnati, Ohio, USA; 3 Division of Biomedical Informatics, Cincinnati Children's Hospital Medical Center, Cincinnati, Ohio, USA; 4 Division of Pathology and Laboratory Medicine, Cincinnati Children's Hospital Medical Center, Cincinnati, Ohio, USA; 5 Division of Biostatistics and Bioinformatics, Department of Environmental and Public Health Sciences, University of Cincinnati College of Medicine, Cincinnati, Ohio, USA; 6 Department of Chemistry, Agilent Metallomics Center of the Americas, University of Cincinnati College of Arts and Science, Cincinnati, Ohio, USA; 7 Department of Pharmacology and System Biology, University of Cincinnati College of Medicine, Cincinnati, Ohio, USA; 8 Department of Electrical Engineering and Computer Science, University of Cincinnati College of Engineering and Applied Sciences, Cincinnati, Ohio, USA; 9 Department of Veterans Affairss, Veteran Affairs Medical Center, Cincinnati, Ohio, USA

**Keywords:** LC3C, lysosome, exocytosis, zinc, renal cancer

## Abstract

Microtubule-associated protein 1 light chain 3 gamma (MAP1LC3C or LC3C) is a member of the microtubule-associated family of proteins that are essential in the formation of autophagosomes and lysosomal degradation of cargo. LC3C has tumor-suppressing activity, and its expression is dependent on kidney cancer tumor suppressors, such as von Hippel–Lindau protein and folliculin. Recently, we demonstrated that LC3C autophagy is regulated by noncanonical upstream regulatory complexes and targets for degradation postdivision midbody rings associated with cancer cell stemness. Here, we show that loss of LC3C leads to peripheral positioning of the lysosomes and lysosomal exocytosis (LE). This process is independent of the autophagic activity of LC3C. Analysis of isogenic cells with low and high LE shows substantial transcriptomic reprogramming with altered expression of zinc (Zn)-related genes and activity of polycomb repressor complex 2, accompanied by a robust decrease in intracellular Zn. In addition, metabolomic analysis revealed alterations in amino acid steady-state levels. Cells with augmented LE show increased tumor initiation properties and form aggressive tumors in xenograft models. Immunocytochemistry identified high levels of lysosomal-associated membrane protein 1 on the plasma membrane of cancer cells in human clear cell renal cell carcinoma and reduced levels of Zn, suggesting that LE occurs in clear cell renal cell carcinoma, potentially contributing to the loss of Zn. These data indicate that the reprogramming of lysosomal localization and Zn metabolism with implication for epigenetic remodeling in a subpopulation of tumor-propagating cancer cells is an important aspect of tumor-suppressing activity of LC3C.

Lysosomes function as the final organelles destined to degrade cargo delivered by autophagosomes and multivesicular bodies. They occupy primarily a perinuclear region of the cell in proximity to microtubule-organizing centers. However, lysosomes can also move away from the nucleus in response to cancer driver events, environmental factors, and nutritional conditions, including amino acid availability, hypoxia, or cholesterol load (1). An important activity of the peripherally located lysosomes is the secretion of their content into the extracellular environment, a process called lysosomal exocytosis (LE) (2). During LE, lysosomes translocate from their perinuclear localization to the proximity of cell surface *via* the microtubule-dependent motor protein kinesin (1). Then they fuse with the plasma membrane in a Ca^2+^-dependent manner, release their luminal content to the exterior of the cell, and expose the luminal site of their integral lysosomal proteins, such as lysosomal-associated membrane protein 1 (LAMP1), on the extracellular surface of the plasma membrane (3). LE is transcriptionally regulated by transcription factor EB (TFEB), a master regulator of genes in lysosomal function (4). TFEB stimulates expression of genes involved in the tethering and docking of the lysosome to the plasma membrane, expression of lysosomal transient receptor potential mucopolin 1 (MCOLN1) channel, which releases lysosomal calcium triggering the final fusion (5). LE allows for cellular clearance and detoxification, including toxic levels of metals (5, 6, 7), and excretion of active proteases that remodel extracellular matrix and contribute to cell motility (8, 9). LE is necessary for membrane repair and remodeling that occur during phagocytosis and neurite outgrowth (10, 11). While release of exosomes is usually attributed to the multivesicular bodies and their fusion with the plasma membrane, LE can also release certain types of exosomes (12). Thus, LE affects intercellular communications in multiple ways.

LE plays an important role in cancer, promoting cancer progression because of the remodeling of extracellular matrix and modulation of tumor cell invasion, angiogenesis, and metastasis. LE was shown to play a role in progression of sarcomas and gliomas (9, 13). In particular, high levels of oversialylated LAMP1 promote LE, a process that can be reversed by the activity of neuraminidase 1, a sialidase that prevents oversialylation of LAMP1 (13). LE plays also important role in chemoresistance (14, 15). Lysosomal sequestration of chemotherapeutics induces LE, preventing them from reaching their intracellular targets (15).

Microtubule-associated protein 1 light chain 3 gamma (LC3C) is a paralog in the microtubule-associated protein light chain family of LC3 autophagic regulators that also includes LC3B and LC3A. LC3s become lipidated on the C-terminal glycine, which allows for the insertion into the elongating membrane of the forming autophagosome and tethers the cargo receptors bringing ubiquitinated cargo to the autophagosomes. LC3C autophagy is an evolutionarily late program present only in higher primates, including humans. LC3C differs in amino acid sequence with other paralogs by 60% and contains a 20 amino acid long C-terminal peptide located after glycine 126, which undergoes lipidation, absent in other paralogs. LC3C forms separate vesicles from LC3A and LC3B paralogs supporting different functions (16). In clear cell renal cell carcinoma (ccRCC), LC3C acts as a tumor suppressor, whereas LC3B has oncogenic activity (17). LC3C expression is suppressed in ccRCC because of the transcription repression by hypoxia-inducible transcription factors (17). Reconstitution of von Hippel–Lindau protein (VHL) in RCC cell lines with lost *VHL* brings back the LC3C, and subsequent LC3C knockdowns lead to tumor formation despite the presence of VHL (17). One of the tumor-suppressing activities of LC3C is inhibition of the accumulation of postdivision midbodies, markers of cancer cell stemness, indicating that the tumor-suppressing activity of LC3C may regulate cancer stem cells (16). LC3C was also shown to have tumor-suppressing activity in breast cancer, where it contributes to degradation of Met receptor tyrosine kinase and regulates its downstream signaling and cell migration and invasion (18). Recently, we reported that LC3C autophagy is regulated by the noncanonical preinitiation and initiation complexes that include Unc-51 like kinase 3, UV radiation resistance–associated gene, Rubicon and PI3K catalytic subunit type 2 alpha (16).

Here, we show that loss of LC3C results in the translocation of lysosomes toward the cell periphery and activation of LE in a subset of RCC cells with reconstituted VHL. This process does not involve autophagic activity of LC3C. Transcriptomic comparison of isogenic cells with low and high LE demonstrate robust reprogramming related to the activity of polycomb repression complex 2 (PRC2), major decrease in the intracellular zinc (Zn), and altered expression of Zn-related genes. Metabolomic analysis revealed also major changes in amino acid steady-state levels. Cells with augmented LE show tumor initiation properties and form aggressive tumors in xenograft models. Immunocytochemistry identified high levels of LAMP1 in the plasma membrane of cancer cells in human ccRCC and reduced levels of Zn, an indication that LE is a frequent event in ccRCC, potentially contributing to the loss of Zn. Overall, these data indicate that an important tumor-suppressing but not autophagic activity of LC3C is regulation of lysosomal activity and Zn metabolism, suggesting epigenetic remodeling of tumor-propagating properties in a subpopulation of cancer cells.

## Results

### Loss of LC3C induces LE in autophagy-independent manner

**Figure 1:**
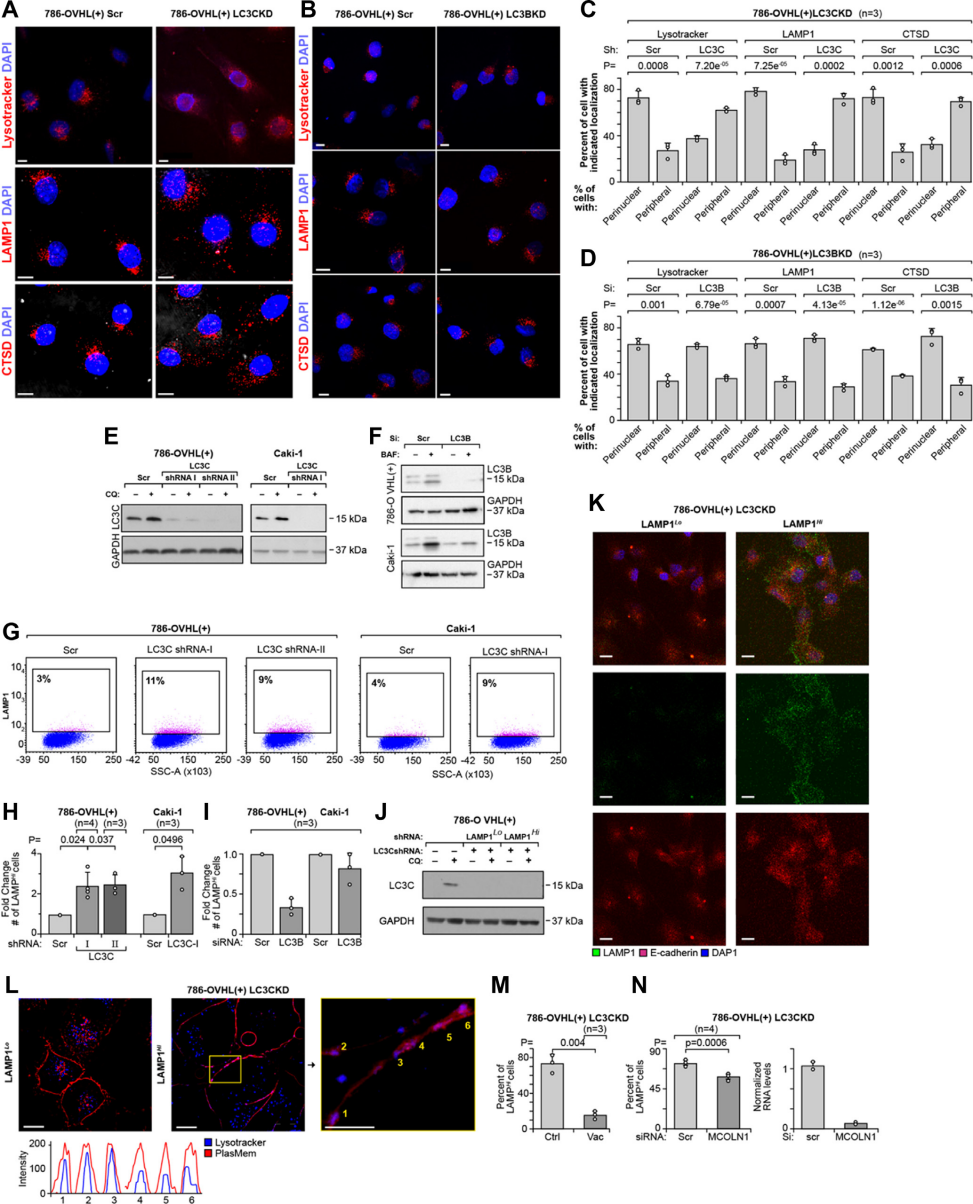
**Loss of LC3C induces cell surface accumulation of LAMP1.***A* and *B*, immunofluorescence for LysoTracker Red, LAMP1, and CTSD in 786-O VHL(+) cells with LC3C-KD (*A*) and with LC3B-KD (*B*). Scale bars represent 10 μm. *C* and *D*, quantification of perinuclear *versus* peripheral distribution of lysosomes using above markers in indicated cells. *E*, Western blot showing knockdown of LC3C in the indicated cell lines. *F*, Western blots showing LC3B knocked down in the indicated cell lines. *G*, scatter plots showing increased number of cells with surface localization of LAMP1 in response to LC3C-KDs in the indicated cell lines. *H*, quantification of enrichment for cells with LAMP1 localized to plasma membrane in the indicated cell lines with LC3C knocked down. *I*, quantification of LAMP1 in plasma membrane in cells with LC3B knocked down. *J*, Western blot shows the same LC3C knockdown in LAMP1^*Lo*^ and LAMP1^*Hi*^ cells. *K*, immunofluorescence of LAMP1 (*green*) and E-cadherin (*red*) on the surface of nonpermeabilized cells in LAMP1^*Lo*^ and LAMP1^*Hi*^ cells. Scale bars represent 10 μm. *L*, live image of the distribution of lysosomes labeled with LysoTracker *Blue* and their colocalization with plasma membrane stained with PlasMem *Bright Red* in LAMP1^*Lo*^ and LAMP1^*Hi*^ cells. RGB profiles are shown. Scale bars represent 10 μm and 5 μm (*inset*). *M*, flow cytometry quantification of cells with LAMP1 localized to plasma membrane in cells treated with vacuolin-1 (Vac, 5 μM) for 24 h. *N*, flow cytometry quantification of cells with LAMP1 localized on the plasma in cells with MCOLN1 knockdown (*left*). Effectiveness of MCOLN1 knockdown was determined by RT–PCR (*right*). *p* Values obtained by two-tailed *t* test in *C*, *D* and *M*; by one-sample *t* test in *H* and by paired two-tailed *t* test in *N*. CTSD, cathepsin D; LAMP1, lysosomal-associated membrane protein 1; LC3C, microtubule-associated protein 1 light chain 3 gamma.

We previously established that reconstitution of VHL in RCC cells with lost *VHL* (*i.e.*, VHL(+) cells) leads to activation of LC3C expression (17). Knockdowns of LC3C, but not LC3B, in 786-OVHL(+) cells induced significant redistribution of lysosomes, labeled with LysoTracker, a marker of mature lysosomes, or stained for lysosomal membrane protein, LAMP1, or aspartic protease cathepsin D (CTSD), from the perinuclear region toward plasma membrane (Fig. 1, *A*–*F*). This result is consistent with the tumor-suppressing activity of LC3C, as cancer cells show a shift toward peripheral positioning of lysosomes to promote tumor invasiveness by secreting lysosomal content to the extracellular environment (19). The result implies increased LE in cells with LC3C knockdown. LE requires Ca^2+^-dependent fusion of lysosomes with plasma membrane exposing luminal domain of LAMP1 on the plasma membrane surface. The presence of this luminal domain can be identified on the surface of live cells using anti-LAMP1 H4A3 antibody, specific for the luminal epitope (3). Using flow cytometry, we measured significant increase in the number of live cells with cell surface localization of LAMP1 in 786-O VHL(+) and Caki-1 cells with LC3C but not LC3B knockdown (Fig. 1, *G*–*I*). Next, we sorted cells with stable LC3C-KD to separate cells marked by high levels of surface LAMP1 (LAMP1^*Hi*^) from those with low levels (LAMP1^*Lo*^). Both cells maintained the same level of LC3C knocked down (Fig. 1*J*). Consistently with the flow cytometry results, immunofluorescent labeling of LAMP1 on live cells without permeabilization and before fixation to determine the presence of LAMP1 on external surface of plasma membrane showed increased fluorescence in LAMP1^*Hi*^ as compared with LAMP1^*Lo*^ cells, whereas the expression of E-cadherin was not different (Fig. 1*K*). Moreover, LAMP1^*Hi*^ cells had an increase in the colocalization of lysosomes labeled with blue LysoTracker and plasma membrane labeled by PlasMem Bright Red in the superresolution microscopy, another indication of LE (Fig. 1*L*). Pretreatment of LAMP1^*Hi*^ cells with vacuolin, an inhibitor of Ca^2+^-dependent LE (20), for 24 h significantly diminished number of cells with LAMP1 on plasma membrane (Fig. 1*M*). Similarly, knockdown of MCOLN1, the channel providing Ca^2+^ necessary for the fusion of lysosome with plasma membrane, inhibited accumulation of LAMP1 on cell surface measured by flow cytometry analysis (Fig. 1*N*). Together, these data demonstrate that LC3C regulates lysosomal localization and exocytosis.

**Figure 2:**
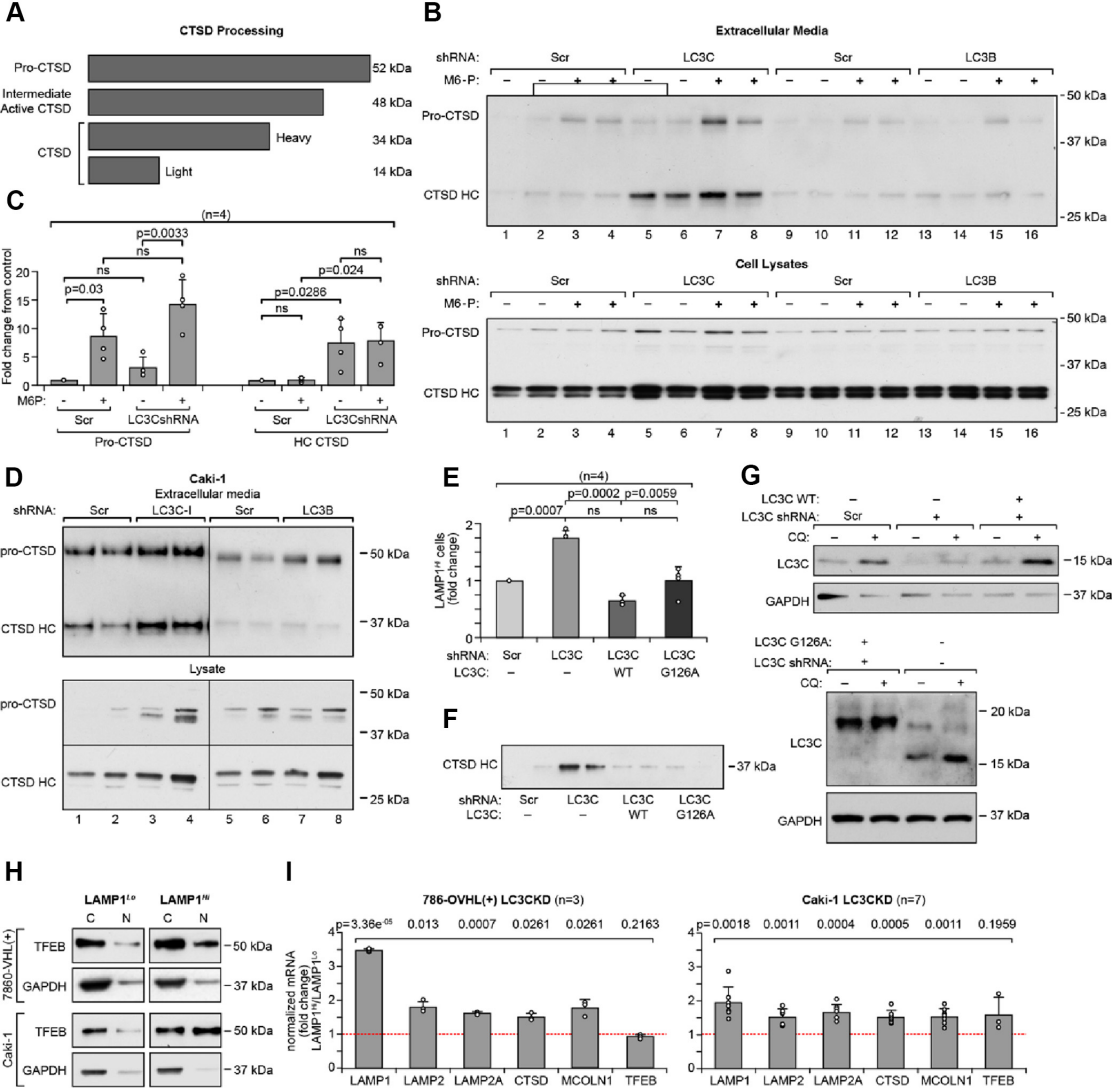
**Loss of LC3C induce LE in autophagy-independent manner.***A*, schematic representation of CTSD proteolytic processing. *B*, Western blots show pro-CTSD and cleaved CTSD heavy chain (HC) in the proteins precipitated from the tissue culture media (*top*) and cellular lysates (*bottom*) in 786-O VHL(+) cell line. Cells were pretreated with 5 mM M6P for 24 h. *C*, quantification of the accumulation of 48 kDa intermediate and cleaved 34 kDa CTSD in response to M6P and indicated knockdowns. *D*, Western blots show pro-CTSD and cleaved CTSD HC in the proteins precipitated from the tissue culture media (*top*) and cellular lysates (*bottom*) in Caki-1 cell line. *E*, effects of re-expression of LC3C WT or autophagy-deficient mutant (G126A) in cells with LC3C-KD on the number of cells with plasma membrane localization of LAMP1. *F*, effects of re-expression of the LC3C on accumulation of CTSD HC in extracellular media. *G*, Western blots show expression of exogenous LC3C similar to the expression of endogenous LC3C. *H*, Western blots show translocation of TFEB from the cytosolic to the nuclear fraction. Each set of Western blots was run together on one gel and quantified from the same exposure. *I*, results of qRT–PCR show induction of mRNA expression for several lysosomal proteins in the indicated cell lines. *p* Values obtained by two-tailed *t* test in *C* and *E* and by one-sample *t* test in (*I*). CTSD, cathepsin D; LAMP1, lysosomal-associated membrane protein 1; LC3C, Microtubule-associated protein 1 light chain 3 gamma; LE, lysosomal exocytosis; M6P, mannose 6-phosphate; qRT–PCR, quantitative RT–PCR; TFEB, transcription factor EB.

In support of these subcellular localization data, we determined that functional indicator of LE, that is, accumulation in the media of lysosomal, mature, cleaved CTSD that migrates at 34 kDa was increased in media from cells deficient in LC3C but not LC3B (Fig. 2, *A*–*D*). CTSD is translated as a pre-proenzyme. After the removal of the signal peptide, pro-CTSD is targeted to the *trans*-Golgi network, where it is modified by mannose 6-phosphate (M6P), binds to mannose 6-phosphate receptors (MPR), and traffics within clathrin-coated vesicles for delivery to lysosomes. In cancer cells, pro-CTSD can be secreted to the extracellular environment and taken up *via* MPRs. In lysosomes, cysteine proteases cleave pro-CTSD to its mature forms, light amino terminal domain (14 kDa), and heavy C-terminal domain (34 kDa) (21) (Fig. 2*A*). Importantly, accumulation of pro-CTSD 48 kDa intermediate in the extracellular media was increased by inhibition of MPR with M6P (5 mM, 24 h) (Fig. 2, *B* and *C*) and did not significantly differ between control cells and cells with LC3C knocked down. This indicates that circulating CTSD through the extracellular environment and return *via* binding to the plasma membrane receptors is a mechanism of CTSD transport to the final lysosomal destination in RCC cells, and this process is not regulated by LC3C. In contrast, accumulation of the 34 kDa mature heavy chain of CTSD in the extracellular media was increased in response to LC3C knockdown (Fig. 2, *A*–*D*). This indicates that mature CTSD is not generated by cleavage of the pro-CTSD intermediate in the extracellular environment but has to be delivered directly from lysosomes.

Finally, we found that increased presence of LAMP1 on cell surface (Fig. 2*E*) and release of active CTSD into the extracellular media (Fig. 2*F*) measured in cells with LC3C-KD were reversed by re-expression of wildtype LC3C and also the autophagy-deficient LC3C mutant, G126A (Fig. 2*G*). This indicates an important autophagy-independent function of LC3C in trafficking of lysosomes to the plasma membrane.

LE is induced at the transcriptional level by the master transcription factor, TFEB, that induces expression of a signature of lysosomal and autophagic genes (4). Consistently, we found nuclear translocation of TFEB (Fig. 2*H*) and significantly increased gene expression for LAMP1, LAMP2, LAMP2A, CTSD, and MCOLN1 in LAMP1^*Hi*^ as compared with LAMP1^*Lo*^ cells (Fig. 2*I*). These data indicate induction of lysosomal biogenesis in cells with LC3C KDs with enriched LE.

### High LE activity corresponds with transcriptional responses to reduced Zn content

**Figure 3:**
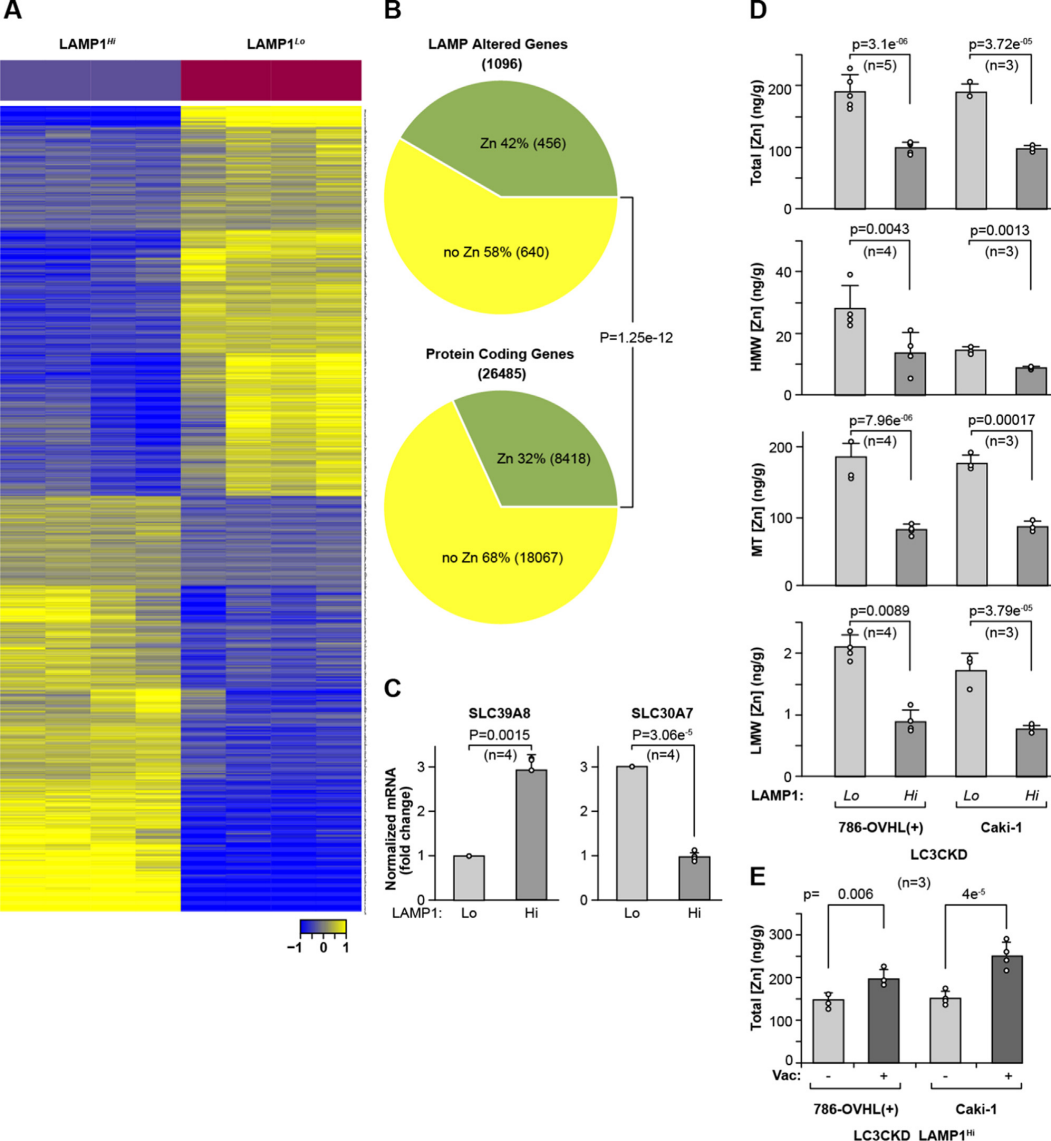
**Cells with high LE show altered expression of Zn-related genes and decreased levels of Zn in several molecular fractions.***A*, heatmap shows differential gene expression between LAMP1^*Hi*^ and LAMP1^*Lo*^ cells. *B*, pie chart shows significant enrichment for Zn-related genes among genes differentially expressed between LAMP1^*Hi*^ and LAMP1^*Lo*^ cells as compared with the number of Zn-related genes among all protein-coding genes in RefSeq database. *p* Value from Chi-square proportions test. *C*, mRNA expression of two Zn transporters SLC39A8 and SLC30A7 in LAMP1^*Lo*^ and LAMP1^*Hi*^ 786-O VHL(+)LC3CKD cells. *p* Value from one-sample *t* test. *D*, measurement of the Zn concentration in total cellular lysates (*top*) and in the high molecular weight fraction (HMW), metallothioneins (MTs), and low molecular weight fraction (LMW) in LAMP1^*Lo*^ and LAMP1^*Hi*^ cells. *E*, inhibition of LE using vacuolin (Vac, 5 μM) rescues intracellular Zn concentration. Zn levels were normalized to cell mass determined by total phosphorus content (63). In *D* and *E*, *p* values were obtained by two-tailed *t* test. LAMP1, lysosomal-associated membrane protein 1; LE, lysosomal exocytosis; Zn, zinc.

In order to determine the molecular effects triggered by LE, we performed RNA-Seq analysis of RNA extracted from four biological replicates of LAMP1^*Hi*^ and LAMP1^*Lo*^ cells isolated by fluorescence-activated cell sorting (FACS). Unsupervised clustering using differentially expressed genes and Pearson correlation–based distance measure clearly stratified the samples by LAMP1 expression (Fig. 3*A*). We found 1096 genes expressed at twofold difference and false discovery rate (FDR) <0.01 (Table S1). About 566 genes were upregulated and 530 genes were downregulated. There was a significant enrichment for the Zn-related genes among genes differentially regulated in LAMP1^*Hi*^ and LAMP1^*Lo*^ cells as compared with the proportion of these genes in RefSeq database (Fig. 3*B* and Table S2). Of 456 Zn-related genes that were differentially expressed, 81 were Zn finger transcription factors. Of those, the expression of 64 was decreased and expression of 17 was increased in LAMP1^*Hi*^ cells. Importantly, two differentially expressed genes are established Zn transporters regulating Zn homeostasis: LAMP1^*Hi*^ cells have increased expression of SLC39A8 (ZIP8), an electroneutral transporter in plasma membrane that regulates uptake of Zn, and decreased expression of SLC30A7 (ZNT7), which facilitates transport of Zn from cytosol to *trans*-Golgi network (Fig. 3*C*). Direct measurement of intracellular Zn using size-exclusion chromatography coupled with inductively coupled plasma mass spectrometry (SEC-ICP-MS) revealed decreased total Zn content as well as diminished Zn content in all molecular fractions (Fig. 3*D*). There was a decrease in Zn bound to proteins, corresponding to high molecular weight fraction, to metallothioneins (MT), small cysteine-rich proteins that buffer metals, and Zn present in the low molecular weight fraction in LAMP1^*Hi*^ cells (Fig. 3*D*). This is likely related to the function of lysosome serving as Zn storage and the role of LE as a mechanism for Zn excretion (7, 22). Importantly, inhibition of LE with vacuolin partially reversed decrease in intracellular Zn (Fig. 3*E*). This indicates that LE leads to a global reprogramming of cellular Zn pools.

### LE leads to Zn-dependent epigenetic reprogramming

**Figure 4:**
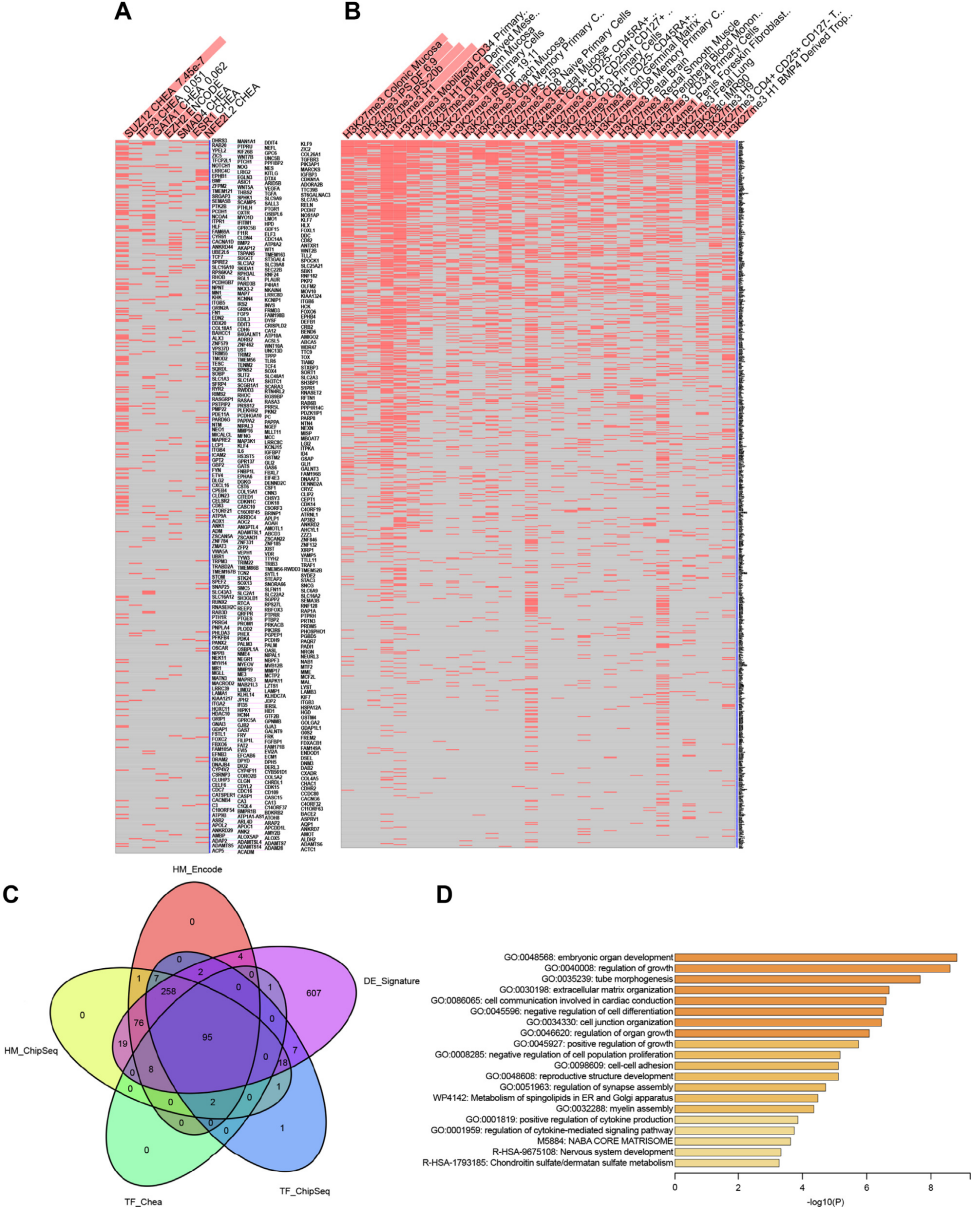
**Analysis of transcriptional regulators common for the altered Zn-related genes shows enrichment for components of PRC2 complex.***A*, Enrichr analysis using ENCODE and CHEA database shows enrichment for SUZ12 and EZH2 transcription factors among genes differentially expressed in LAMP1^*Lo*^ and LAMP1^*Hi*^ 786-O VHL(+)LC3CKD cells. *B*, Enrichr analysis using Epigenomics Roadmap HM ChiP-Seq database shows enrichment for H3K27Me3 for genes differentially expressed in LAMP1^*Lo*^ and LAMP1^*Hi*^ 786-O VHL(+)LC3CKD cells. *C*, Venn diagram identifies 95 genes differentially expressed in LAMP1^*Lo*^ and LAMP1^*Hi*^ 786-O VHL(+)LC3CKD cells and determined to be regulated by SUZ12/EZH2 and H3K27Me3 in the indicated databases. *D*, top 20 pathways significantly enriched for the set of 95 differentially expressed genes using Metascape analysis. LAMP1, lysosomal-associated membrane protein 1; PRC2, polycomb repression complex 2; Zn, zinc.

Analysis of transcription factors related to the genes differentially regulated between in LAMP1^*Hi*^ and LAMP1^*Lo*^ cells in Enrichr web server (23, 24, 25) revealed a significant enrichment for binding sites specific for the SUZ12 member of PRC2 using ENCODE and CHEA Consensus TFs from Chip-X database (Fig. 4*A*) (26) and for histone 3 K27 trimethylation using Epigenomics Roadmap HM chromatin immunoprecipitation (ChiP)-Seq database (Fig. 4*B*). This is consistent with PRC2 activity as H3K27 methyltransferase maintains epigenetic repression of silenced genes. Importantly, 95 genes upregulated in LAMP1^*Hi*^ cells were associated with SUZ12 and EZH2 in ENCODE and ChEA Consensus TF and ENCODE TF ChIP-Seq 2015 and with H3KMe3 in ENCODE Histone Modifications 2015 and Epigenomics Roadmap HM ChIP-Seq databases (Fig. 4*C* and Table S3). The most significant pathways enriched among these genes was Epithelial Mesenchymal Transition (MSigDB Hallmark 2020) and regulation of development, morphogenesis, differentiation, and extracellular matrix organization (Metascape, (27)) (Fig. 4*D*).

**Figure 5:**
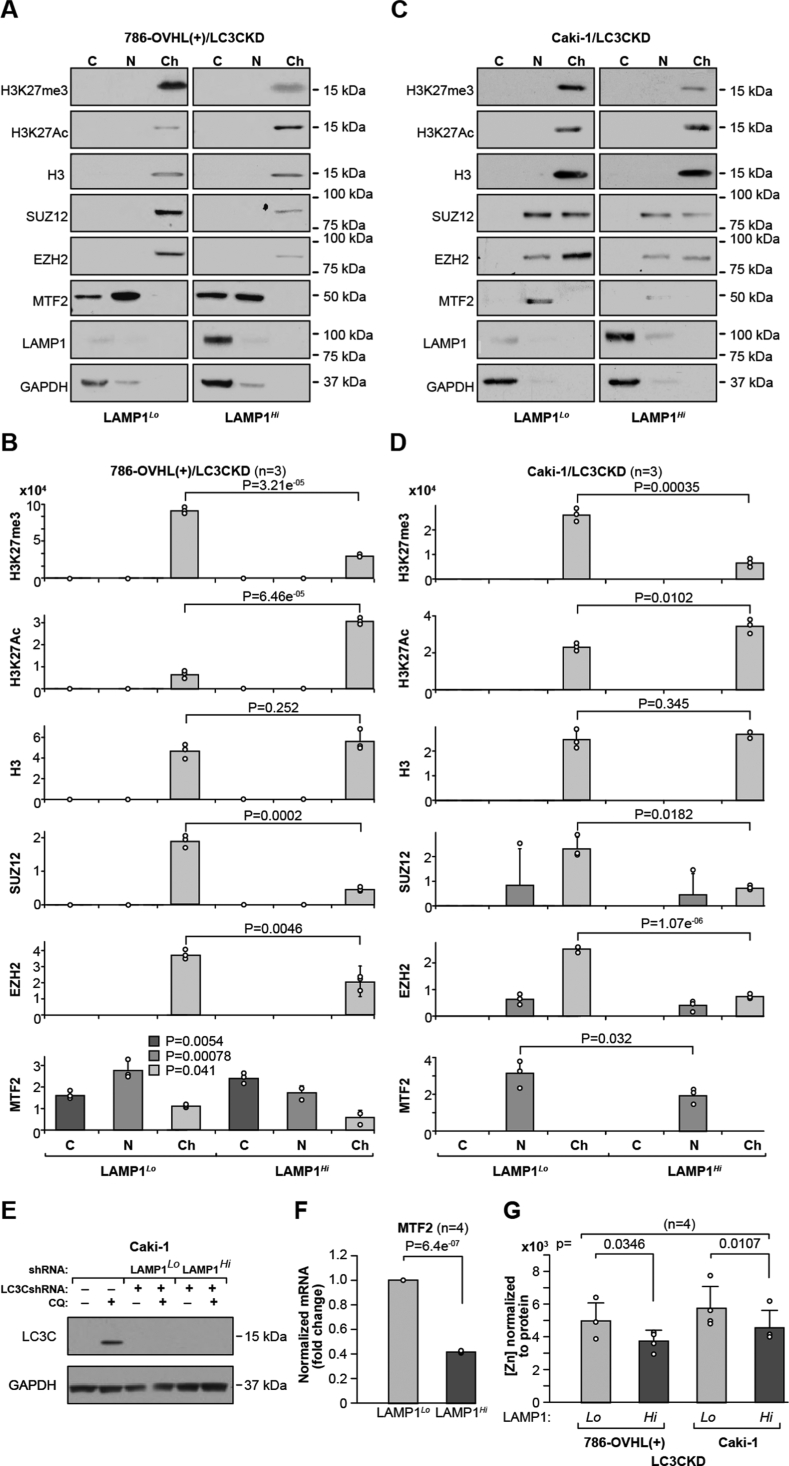
**Cells with increased LE show diminished H3K27me3 and decreased presence of PRC2 components in chromatin fraction**. *A* and *B*, Western blots and (*C* and *D*) respective quantification for H3K27me3, H3K27Ac, and indicated components of PRC2 complex. Each set of Western blots was run together on one gel and quantified from the same exposure. *p* Values obtained from two-tailed *t* test. *E*, Western blot showing similar level of LC3C knock down in LAMP1^*Lo*^ and LAMP1^*Hi*^ Caki-1 cells. *F*, expression of MTF2 mRNA expression in LAMP1^*Lo*^ and LAMP1^*Hi*^ cells. *p* Value from one-sample *t* test in (*G*). Zn concentration in the chromatin-enriched fraction as shown in (*A* and *B*). Zn levels were normalized to the total protein content in chromatin fractions. *p* Values were obtained by two-tailed *t* test. LAMP1, lysosomal-associated membrane protein 1; LE, lysosomal exocytosis; PRC2, polycomb repression complex 2; Zn, zinc.

Fractionation of cell lysates into nuclear and chromatin fractions revealed overall decrease in H3K27Me3 and components of PRC2, SUZ12, EZH2, and MTF2, while there was an increase in H3K27Ac in LAMP1^*Hi*^ cells (Fig. 5, *A*–*D*). Note that LC3C knockdown is the same in LAMP1^*Hi*^ and Lamp1^*Lo*^ cells (Figs. 1*J* and 5*E*). This indicates major PRC2-related transcriptional reprogramming. PRC2 subunits utilize Zn ions, and SUZ12 has C2H2-type Zn finger motif, essential for its activity (28). EZH2 has a CXC domain with two Zn_3_Cys_9_ motifs in its SET domain (29). Moreover, the polycomb-like (PCL) protein necessary for recruitment of PRC2 to the nucleation sites, PCL2 or MTF2, is also a Zn-finger transcription factor binding to metal-responsive elements regulating expression of the MTF2 gene (30). Consistently, expression of MTF2 mRNA was decreased in LAMP1^*Hi*^ cells (Fig. 5*F*). We also determined that there was a significant decrease in the Zn concentration in the chromatin fraction (Fig. 5*G*), supporting Zn-responsive epigenetic reprogramming.

### Activation of LE leads to metabolomic reprogramming

**Figure 6:**
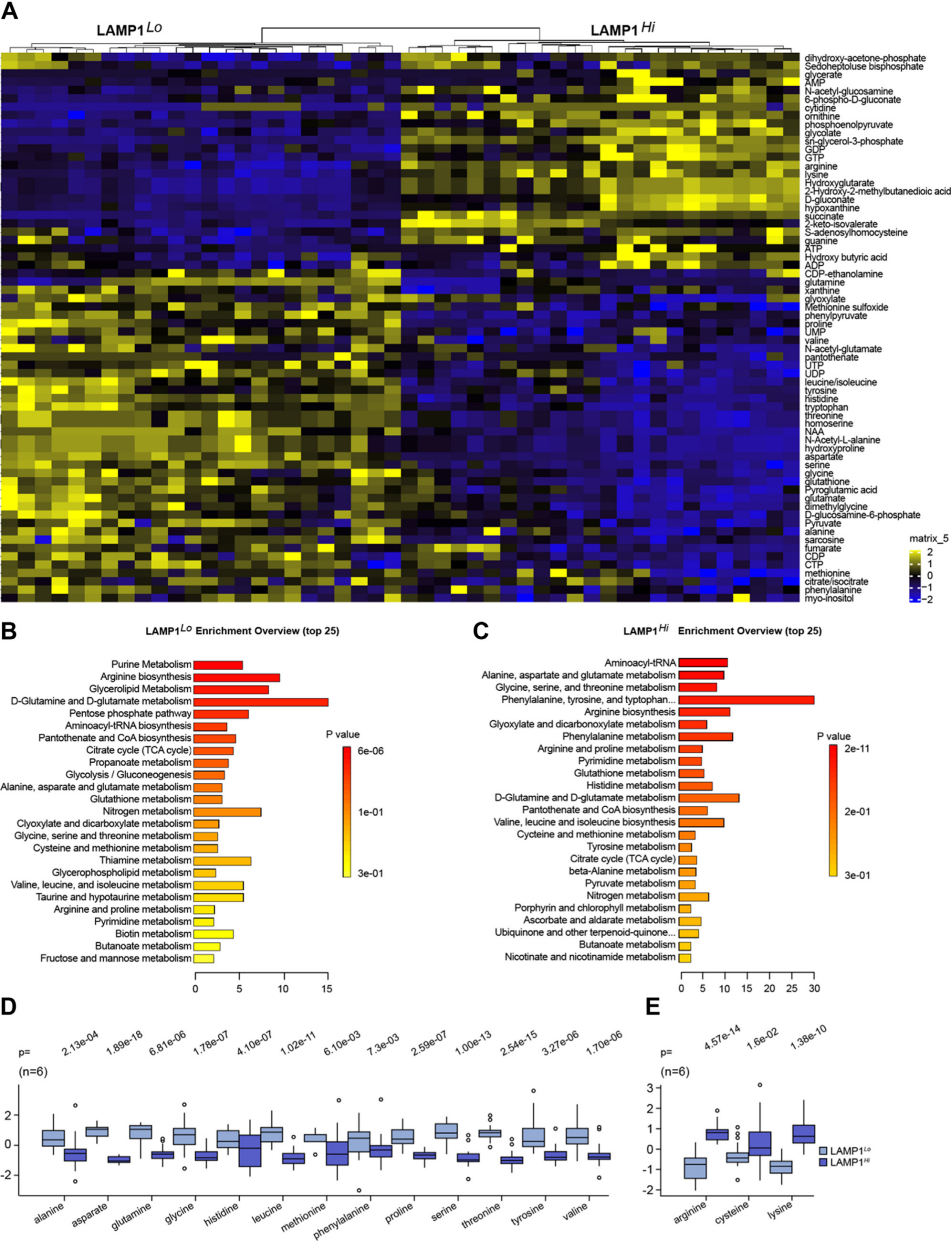
**Metabolomic landscape of cells with high LE shows major changes in steady-state levels of amino acids.***A*, heatmap showing differential abundance of steady-state levels of metabolites in LAMP1^*Lo*^ and LAMP1^*Hi*^ cells. *B* and *C*, identification of metabolic pathways from enriched LAMPL1^*Lo*^ and LAMP1^*Hi*^ cells, respectively. *D*, decreased abundance of several amino acids in LAMP1^*Hi*^ cells. *E*, increased abundance of three amino acids in LAMP1^*Hi*^ cells. *p* Values were obtained by two-tailed *t* test. LAMP1, lysosomal-associated membrane protein 1; LE, lysosomal exocytosis.

Lysosomal activity is a major regulator of cellular metabolism. In order to determine how LE affects metabolic state of cells, we performed LC–MS analysis of steady-state levels of cellular metabolites. The analysis of cellular lysates revealed clear differences in the steady-state levels of 77 metabolites at FDR <0.05 between LAMP1^*Hi*^ and LAMP1^*Lo*^ cells (Fig. 6*A* and Table S4). Levels of 31 metabolites were higher in LAMP1^*Hi*^ cells, whereas levels of 46 metabolites were higher in LAMP1^*Lo*^ cells. There was a highly significant enrichment for pathways related to amino acid metabolism and protein synthesis (Fig. 6, *B* and *C*). Steady-state levels of most amino acids, including glutamate, valine, serine, threonine, tyrosine, histidine, alanine, tryptophan, leucine, glycine, aspartate, proline, glutamine, and methionine were decreased in LAMP1^*Hi*^ cells (Fig. 6*D*). Only levels of three amino acids, arginine, lysine, and cysteine, were increased in LAMP1^*Hi*^ cells (Fig. 6*E*). This overall decrease in amino acid levels in LAMP1^*Hi*^ cells can be attributed to the diminished activity of lysosomes in cellular biodegradation of protein and regeneration of nutrients because of their destination toward releasing cargo into the extracellular environment or changed activity in sensing cellular amino acid levels (31). There was also an increase in abundance of S-adenosylhomocytseine (SAH), a product of methyl transfer reaction from S-adenosylmethionine (SAM), universal donor of methyl group, to the targets (Table S4). These data are consistent with major role of lysosomes in regulation of cellular amino acid content.

### Cells with high LE activity show tumor-initiating properties

**Figure 7:**
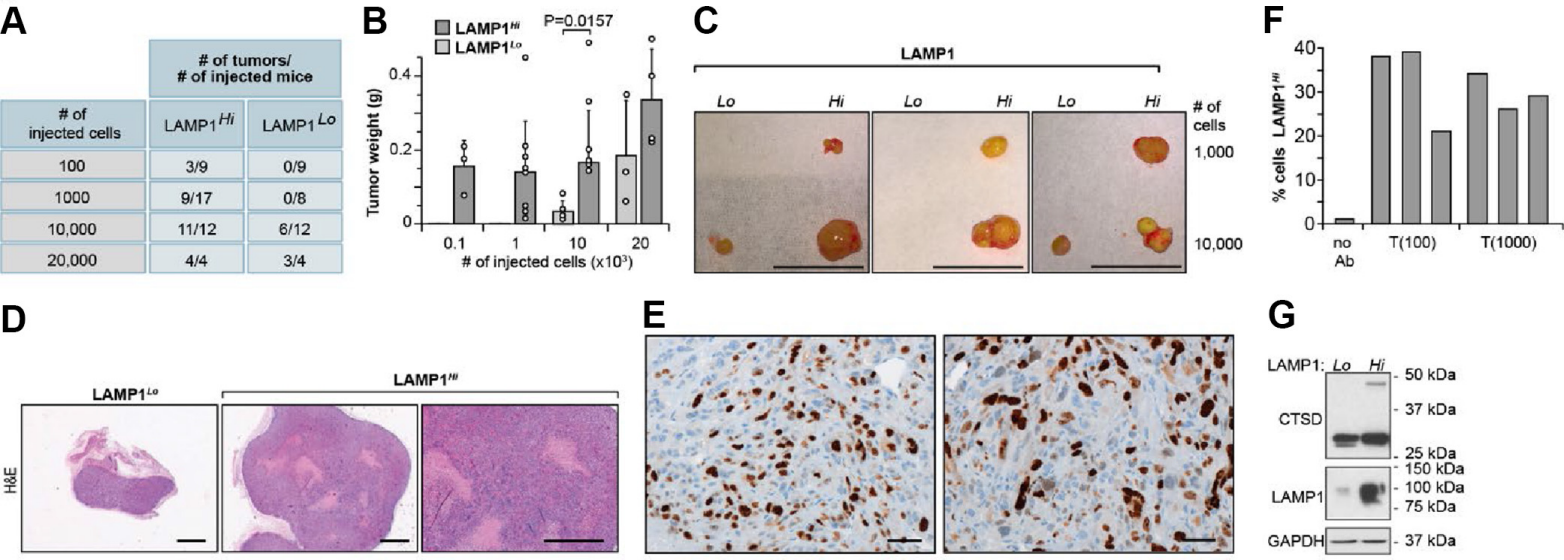
**LAMP1**^***Hi***^**but not LAMP1**^***Lo***^**cells form malignant tumors in xenograft assays.***A*, limited dilution assay: numbers of tumors formed by indicated numbers of injected cells. *B*, tumor weight for the indicated numbers of injected cells. *p* Value from two-tailed *t* test for 10,000 injected cells. *C*, representative examples of tumors formed by the indicated numbers of cells. Scale bar represents 10 mm. *D*, H&E staining of tumors formed by LAMP1^*Lo*^ and LAMP1^*Hi*^ cells. Scale bar represents 50 μm. *E*, immunohistochemical staining for Ki67 on sections from tumors formed by LAMP1^*Hi*^ cells. Scale bar represents 1 mm. *F*, FACS analysis of cells dissociated from three xenograft tumors formed by injection of 100 cells or 1000 cells shows enrichment for cells with LAMP1 present in the plasma membrane. *G*, Western blot of cells isolated from xenograft tumors shows maintained high levels of LAMP1 and CTSD expression. CTSD, cathepsin D; FACS, fluorescence-activated cell sorting; LAMP1, lysosomal-associated membrane protein 1.

We have previously shown that knockdowns of LC3C in 786-O VHL(+) and A498 VHL(+) cells induced tumor formation in orthotopic xenografts (17). To determine whether regulation of LE mediates tumor-suppressing effects, we tested tumor formation and growth in mice injected with LAMP1^*Hi*^ and LAMP1^*Lo*^ cells in a limiting dilution assay. Clearly, cells with high level of LE showed a significantly higher number of tumors at lower number of injected cells, and these tumors were significantly larger than as compared with tumors formed by LAMP1^*Lo*^ cells (Fig. 7, *A*–*C*). The tumors were histologically malignant, with large areas of necrosis as shown in the H&E staining (Fig. 7*D*) and strong labeling of the nuclei with proliferation marker, Ki67 (Fig. 7*E*). The tumor cells maintained high levels of LAMP1 in the plasma membrane as was determined by FACS analysis of cells isolated from individual tumors (Fig. 7*F*), and Western blot shows higher levels of LAMP1 and CTSD in cells isolated from tumors formed by LAMP1^*Hi*^ cells (Fig. 7*G*).

**Figure 8:**
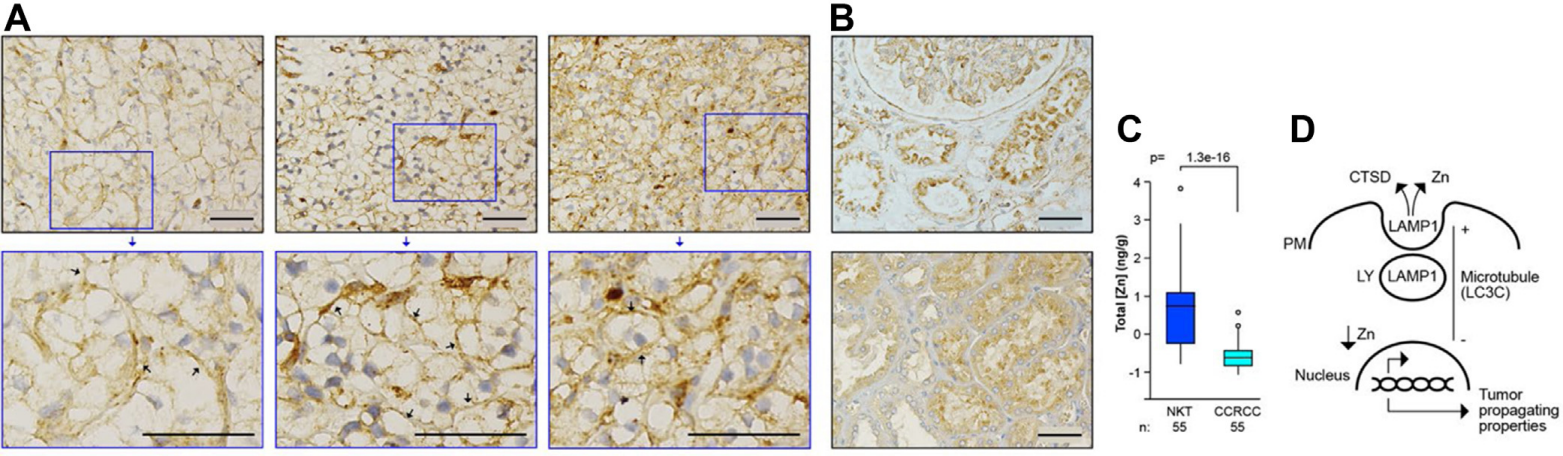
**Human ccRCC shows plasma membrane localization of LAMP1 in cancer cells and reduced Zn levels as compared with normal kidney tissues.***A*, immunohistochemical staining for LAMP1 in sections of three ccRCCs. Scale bars represent 50 μm. *Arrows* point to plasma membrane localization of LAMP1 cells in cancer cells. *B*, immunohistochemical staining for LAMP1 in sections of two normal kidney tissues. *C*, low level of total Zn in ccRCC (n = 55) as compared with normal kidney tissues (n = 55). Zn content was normalized to total tissue mass measured by phosphorus content (63). Data were log_2_ normalized. *p* Value was obtained by two-tailed *t* test. *D*, model of proposed regulation. ccRCC, cancer cell in human clear cell renal cell carcinoma; LAMP1, lysosomal-associated membrane protein 1; LY, lysosome; PM, plasma membrane; Zn, zinc.

Analysis of human ccRCC revealed plasma membrane localization of LAMP1 on cancer cells (Fig. 8*A*), whereas kidney epithelial cells showed cytoplasmic localization (Fig. 8*B*). Moreover, analysis of Zn levels revealed significantly lower levels of Zn in ccRCC as compared with the normal kidney tissue (Fig. 8*C*). These data indicate that LE could contribute to lower Zn level and Zn-related epigenetic modifications in ccRCC.

## Discussion

Here, we report that peripheral positioning of the lysosomes and LE is regulated by LC3C, and this regulation does not depend on LC3C autophagic activity. Lysosomal movement between perinuclear region and cell periphery requires microtubules and kinesin and dynein motors for anterograde and retrograde transport, respectively (1). We propose that LC3C activity as a microtubule-associated protein inhibits peripheral localization of lysosomes, consistently with LC3C role as a tumor suppressor. Peripheral localization of lysosomes promotes LE leading to remodeling of the extracellular matrix and supporting migration and invasion and increases lysosome association with mammalian target of rapamycin (mTOR) supporting tumor growth (1). Because lysosomes serve as Zn storage (7, 22), extracellular release of Zn during LE results in alterations in Zn homeostasis and causes transcriptional, epigenetic, and metabolic reprogramming (Fig. 8*D*). Interestingly, while the peripheral localization of lysosomes is a robust response to LC3CKD, only a subpopulation of cells shows induction of LE and resulting remodeling associated with formation of fast-growing tumors. This reveals a novel subpopulation of RCC cells with tumor initiating/propagating properties related to LE enriched by loss of LC3C and is also consistent with previous work showing the role of LC3C in regulation of “cancer stem cell–like properties” (16).

Zn is an important trace element with several tumor growth–regulating activities (32, 33). It is required for the activity of transcription factors and enzymes containing Zn-finger domains that control transcription and DNA repair, including p53. It has antioxidative properties by regulating expression of metal-binding proteins, metallothioneins, and Cu/Zn superoxide dismutase. It regulates cell proliferation and apoptosis. In addition, Zn is likely to affect tumor microenvironment by modulating tumor immunity and inflammation (34). Serum levels of Zn are decreased in several cancers (35). Zn has tumor-suppressing activities, and its levels are decreased in several solid tumor cancers, including prostate (36, 37), bladder (38), liver (39), bone (40) and head and neck (41). However, in breast cancer, Zn contributes to cancer progression (42). Levels of Zn are regulated by the expression of Zn transporters involved in its uptake (43). Our work indicates that LE is a powerful mechanism modifying the levels and functions of intracellular Zn and induces adaptive changes in expression of Zn transporters essential for maintaining its homeostasis.

Importantly, Zn supplementation appears to have positive therapeutic effect in several *in vivo* animal studies and was suggested in management and chemoprevention of cancer in patients (44). So far information regarding Zn levels in ccRCC has been limited. The low levels of Zn in ccRCC indicate this cancer as a potential candidate for dietary Zn supplementation.

Changes in Zn likely affect numerous transcription events as 81 Zn-finger transcription factors have altered expression and Zn-finger transcription factors are established transcriptional regulators in cancer (45). Variations in Zn level are also known to cause extremely long-lasting epigenetic modifications, including prenatal exposures to Zn that epigenetically regulate gene expression in adults (46, 47, 48). In particular, Zn deficiency has been shown to decrease histone H3K4 trimethylation and global DNA methylation (48). Here, we show that decreased levels of cellular and chromatin fraction of Zn and change in the expression of several Zn-regulated proteins correlate with diminished chromatin association of members of PRC2 complex and histone H3K27 trimethylation. The core subunit of PRC2 complex, SUZ12, as well as accessory subunit, MTF2, are Zn-finger proteins. Interestingly, MTF2 was originally described as metal-responsive transcription factor, which was able to bind to the metal-responsive elements in a Zn-dependent manner in yeast (30). While ultimately MTF2 was determined to be PCL2 member of PRC2 complexes and to modulate H3K27 methylation, little is understood about the possibility that it could serve as a Zn sensor and translate Zn availability into epigenetic modification regulating gene expression. Induction of SLC39A8 (ZIP8) transporter in LAMP1^*Hi*^ cells is likely an adaptive mechanism in response to the decrease in cellular Zn content. However, this transporter facilitates uptake of other ions (including Mn, Fe, Cd, and Se), thus its augmented expression can promote signaling pathways activated by these metals that can contribute to cancer progression (49, 50, 51).

The activities of PRC2 are both oncogenic and tumor suppressing (52). Low H3K27 methylation, including H3K27me1, H3K27me2, and H3K27me3, is a negative prognostic factor in ccRCC (53, 54), which would be consistent with our results showing decreased H3K27me3 in cells with high LE that form highly malignant tumors. Clearly, final H3K27 methylation is a readout of combined activities of methyltransferases, demethylases, and overall metabolic landscape contributing to the generation of methyl groups. Here, it can be proposed that this epigenetic footprint is regulated by Zn dyshomeostasis.

We found robust metabolic reprogramming related to high LE with potential consequences for epigenetic modifications. The predominant feature, decreased steady-state levels of several amino acids, can be related to altered lysosomal processes, such as degradation of intracellular and extracellular cargos. Interestingly, only steady-state levels of lysine, arginine, and cysteine are increased. Arginine and lysine are positive regulators of mTORC1 (55, 56) and their increase may be important for the maintenance of mTORC1 activity when levels of leucine, another amino acid promoting mTORC1 activity (57), are diminished. Therefore, increased LE may contribute to a switch in the mechanisms regulating mTORC1 and anabolic activity. Increased cysteine levels are indicative of potential effects of LE on the sulfur metabolism and redox potential. The amino acid reprogramming can be also causative for the changes in histone methylation. First, there is a decrease in the level of methionine, and methionine levels can affect H3K4me3 (58). There is also upregulation of SAH in LAMP1^*Hi*^. SAM, the essential source of methyl groups, is synthesized from methionine and ATP by methionine adenosyltransferase. The product of methyltransferase reaction, SAH, inhibits methyltransferase activity. Thus, small fluctuations in SAM and SAH levels and a decrease of the SAM/SAH ratio can diminish histone methylation at H3K9me2 and H3K27me3 (59). Serine and glycine contribute one carbon units for SAM biosynthesis either by procurement of ATP or methionine recycling (60). Other amino acids, such as threonine, have been shown to be indirectly essential for SAM synthesis and H3K4 methylation in mouse embryonic stem cells (61).

Altogether, the data demonstrate a novel nonautophagic function of LC3C regulating lysosomal positioning and LE, with functional consequences for Zn-dependent regulation of transcription, metabolic rewiring, and tumor formation.

## Experimental procedures

### Cell culture, treatments, transfection, and lentiviral transductions

Human RCC cell lines, 786-O with reconstituted VHL (786-O VHL(+)) and Caki-1 with endogenous VHL were used. Both cells express high levels of LC3C (16). Stable LC3C knockdowns were performed using GIPZ shRNAs in lentiviral vectors, whereas plko-shRNAs or siRNAs were used for LC3B and MCOLN1 knockdowns (Dharmacon) (16, 17). For the reconstitutions of LC3C wildtype or G126A mutant, exogenous LC3C was expressed from the lentiviral construct pLX304 (CCSB-Broad Lenti ORF MAP1LC3C) (16). Lentiviral DNA constructs were VSV-G envelope packaged. Cells were grown in Dulbecco's modified Eagle's medium and HAM's F-12 (DMEM/F12) (Hyclone SH30023) with 10% fetal bovine serum (FBS). All cell analyses were performed 48 h from the original plating. Treatments with vacuolin (5 μM) or M6P (5 mM) were performed for 24 h before collection. RNA extractions and quantitative RT–PCR (qRT–PCR) were described before (17). Primer sequences are listed in Table S5.

### Flow cytometry determination of surface LAMP1

Cells were trypsinized, washed with PBS, and incubated in DMEM containing 1% FBS and 1% bovine serum albumin (BSA) with Alexa Fluor 647 Mouse Anti-Human LAMP1 Clone H4A3 (BD Biosciences), which detects intraluminal epitope of LAMP1, for 1 h at 4 °C with intermittent mixing. Cells were washed in PBS and resuspended in PBS with 1% BSA. Control cells were treated the same way, excluding incubation with the primary anti-LAMP1 antibody or with isogenic antibodies (Alexa Fluor 647 mouse IgG1K isotype; BD Biosciences, catalog no.: 557732). Xenograft tumors were dissected and minced, following by incubation in DMEM/F12 media with 5% FBS, 20 μg/ml gentamycin, 300 units/ml collagenase, and 100 units/ml hyaluronidase at 37 °C for 2 to 4 h. Floating cells were collected and resuspended in prewarmed DMEM/F12 with 5% FBS and 2 units/ml DNAse I with antibiotics. Cells were incubated with 30 μl LAMP1 ab-Alexa 647 and 4′,6-diamidino-2-phenylindole (1:1000 dilution). Filtered single-cell populations were sorted and analyzed using a BD FACS Aria III. Allophycocyanin-conjugated LAMP1 was measured using the 633-excitation laser and a 660/20 nm emission filter. BD FACS Diva software (version 8.0) was used to run the instrument and to analyze the data. Forward angle scatter, right angle scatter, and fluorescence intensity were recorded from 50,000 cells. Forward scatter and side scatter used to separate live from dead cells and debris. Cell viability was confirmed using Trypan Blue exclusion assay.

### Immunofluorescence experiments

Cells grown on glass coverslips were fixed with 100% methanol for 5 min at −20 °C. Cells were permeabilized with 0.1% saponin. Coverslips were blocked with PBS containing 0.1% saponin and 1% BSA for 30 min and incubated with primary mouse anti-LAMP1 antibody (Abcam; catalog no.: ab25630, 1:3000 dilution) or rabbit anti-CTSD antibody (Abcam; catalog no.: ab6313, 1:2000 dilution) for 1 h at 37 °C. Coverslips were then washed and incubated with Alexa Fluor-labeled secondary antibodies (anti-mouse AF 555 and anti-rabbit AF568; Invitrogen) for 30 min at room temperature. For determination of surface, LAMP1 cells were incubated the same anti-LAMP1 antibody (1:100 dilution) or E-cadherin (Abcam; catalog no.: ab15148, 1:100 dilution) in PBS with 1% BSA at 4 °C for 30 min. Then cells were washed and fixed using 2% paraformaldehyde and incubated with Alexa Fluor 555 anti-mouse secondary antibodies. Finally, in each case, coverslips were washed and mounted using 4′,6-diamidino-2-phenylindole Fluoromount-G (Southern Biotech). Lysosomes were labeled with LysoTracker Red DND-99 (Invitrogen; catalog no.: L7528) or for live imaging with LysoTracker Blue DND-22 (Invitrogen; catalog no.: L7525). Plasma membrane was labeled with PlasMem Bright Red (Dojind; catalog no.: P505).

Confocal images were acquired on a Zeiss LSM710 confocal microscope with a Zeiss Axio.Observer Z1 stand and a Zeiss Plan-Apochromate objective (63×/1.4 Oil DIC) using the Zeiss Zen2010 software. The appropriate lasers and emission filters for the respective fluorophores were used in a multitracking mode. Widefield images were acquired using a Zeiss Axioplan 2 imaging microscope with the appropriate filter cubes and a Zeiss AxioCam MRm B&W camera to record the images using the Zeiss AxioViosion (Rel4.7) software. The objectives had the following specifications: Plan Apochromat 10/0.45; Plan Neofluar 20×/0.5; Plan Neofluar 40×/0.6 (air); Plan Neofluar 40×/1.3 (Oil); Plan Apochromat 63×/a.4 (Oil): alpha-PlanFluar 100×/1.45. All images were saved as 8 bit images.

Live cells were imaged on a Nikon N-SIM super-resolution microscope on a motorized inverted Nikon Eclipse Ti stand with a Hamamatsu Orca Flash 4.0 camera and SR HP Apo TIRF 100xH objective. Images were acquired and reconstructed using Nikon NIS-Elements AR 5.11.00 software.

Quantification of LAMP1 perinuclear *versus* peripheral distribution was performed as described (62). Cells were considered to have peripheral *versus* perinuclear distribution pattern of lysosomes if more than 50% of LAMP1 staining was in the peripheral *versus* perinuclear region, respectively. Approximately 200 to 400 cells were counted in three independent experiments.

### Extraction of CTSD from the media

Proteins were extracted using acetone precipitation method. Tissue culture media from control and treated cells was incubated with five volumes of acetone (HPLC grade) for 5 h at −20 °C to precipitate proteins, and the pellet was obtained by centrifugation at 15,000*g* for 10 min. The pellets were dried, washed, solubilized, and equal amounts of proteins were used for PAGE.

### Cell fractionation and Western blot analysis

Cells (4 × 10^6^) were washed, centrifuged, and incubated with 5× cell pellet volume of hypotonic buffer A (10 mM Hepes, pH 7.5, 10 mM KCI, 1.5 mM MgCl_2_, 10% glycerol, 1 mM DTT, 0.1% Triton and protease, and phosphatase inhibitors) on ice for 5 min. Cells were centrifuged at 1700*g*, and this step was repeated the second time. The supernatant was collected as cytoplasmic extract. The remaining nuclear pellet was resuspended in 200 μl of high salt buffer B (10 mM Hepes, pH 7.5, 300 mM NaCl, 1.5 mM MgCl_2_, 10% glycerol, 1 mM DTT, and protease and phosphatase inhibitors) and extracted with rotation for 1 h at 4 °C. After centrifugation, supernatant was saved as soluble nuclear extract, and the remaining chromatin/nucleic acid pellet was washed twice with buffer A to remove salt. Then the pellet was resuspended in 200 μl micrococcal nuclease/DNase digestion buffer (50 mM Tris, 50 mM NaCl, 5 mM CaCl_2_, and 2.5 mM MgCl_2_) and sonicated four times for 20 s. Micrococcal nuclease (2 U) and DNAse (37 U) were added, and digestion was performed for 1 h at 37 °C. The reaction was stopped by addition of EGTA to a final concentration of 20 mM and centrifuged at 14,000 rpm for 15 min at 4 °C to obtain chromatin-enriched extract. Cytoplasmic and soluble nuclear extracts were also centrifuged at 14,000 rpm for 15 min at 4 °C. Equal protein amounts of each extract were separated on 10% or 4 to 12% polyacrylamide gels and transferred onto polyvinylidene fluoride membrane. Blots were probed with antibodies against: CTSD (Abcam; catalog no.: ab6313), LC3C (CST; catalog no.: 14736), GAPDH (Abcam; catalog no.: ab8245), TFEB (CST; catalog no.: 4240), H3K27me3 (CST; catalog no.: 9733), H3K27Ac (CST; catalog no.: 4353), histone H3 (Invitrogen; catalog no.: PA5-16183), SUZ12 (CST; catalog no.: 3737), EZH2 (CST; catalog no.: 5246), MTF2 (Avivia; catalog no.: ARP34292 P050), and LAMP1 (Santa Cruz; catalog no.: sc-5570).

### Xenografts

Experiments on mice were performed in accordance with University of Cincinnati Institutional Animal Care and Use Committee–approved protocols. For subcutaneous injections, indicated number of cells in cell culture medium containing 50% Matrigel were injected into the flanks of athymic nude mice. At the end of 12 weeks, mice were sacrificed, and tumors were dissected, weighted, fixed in 4% paraformaldehyde and processed for H&E staining and immunocytochemistry using anti-Ki67, cell proliferation marker (17). Tumors were digested to obtain individual cells, and LAMP1 expression was determined using Western blot and FACS.

### Zn measurements

Cell pellets (0.5 × 10^6^ cells) or chromatin pellets (as described in the aforementioned section) were acid digested with nitric acid in a CEM Discover microwave to reduce the carbon load and to mineralize all compounds associated with the elements of interest. Digested samples (1–5 mg) were diluted with ultrapure water to reduce the acid concentration below 3% and loaded into the ICP-MS–MS (triple quad Agilent 8800x ICP-MS–MS). The instrumental conditions were optimized to remove interferences by using a collision/reaction cell. Integration time was adjusted according to the concentration range for each particular element. Multiple isotopes were monitored when possible to ensure that no interferences were present. The external calibration method was used from 0.01 to 2500 ng ml^−1^ for the elements of interest. A mixture of scandium, yttrium, indium, and bismuth was spiked to the samples and calibration as internal standards at 5 ng ml^−1^ to correct for sensitivity drifts. In order to increase the accuracy, internal mass index elemental tags were used in the form of P and S instead of the sample mass (63). The data analysis was performed with Agilent MassHunter software, with internal standard recoveries and calibration curves. The results are expressed in nanogram of element per gram of sample. Quality control samples used include NIST SRM 2668-Toxic Elements in Frozen Human Urine standard reference material and the NIST Bovine muscle powder SRM 8414.

### RNA analysis

RNA was extracted using RNAlater ICE (Ambion; catalog no.: AM7030) following by miRNA isolation kit (Ambion; catalog no.: AM1560). The quality of RNA isolated was checked using Bioanalyzer RNA 6000 Nano kit (Agilent). For RNA-Seq, polyA RNA was extracted using NEBNext Poly(A) mRNA Magnetic Isolation Module (NEB). RNA-Seq libraries were prepared using NEBNext Ultra II Directional RNA Library Prep Kit (NEB). After library quality control analysis using Bioanalyzer High Sensitivity DNA kit (Agilent) and library quantification using NEBNext Library Quant kit (NEB), the sequencing was performed under the setting of single read 1 × 51 bp to generate ∼30 million reads per sample on HiSeq 1000 sequencer (Illumina). Data analysis was performed as described before (64).

A list of 8418 Zn-related genes was obtained by querying RefSeq (65). The overlap of these genes with the LAMP1^*Hi*^ *versus* LAMP1^*Lo*^ signature was determined (456 genes), and pie charts were generated using R base graphics for both the percentage of Zn-related genes in the LAMP1^*Hi*^ *versus* LAMP1^*Lo*^ signature and the percentage of Zn-related genes in all protein-coding genes. A Chi-square proportions test was performed in R to assess the significance of the proportion of Zn-related genes in the LAMP1^*Hi*^ *versus* LAMP1^*Lo*^ signature, as compared with the proportion of Zn-related genes as a percentage of protein-coding genes.

For qRT–PCR, RNA was purified using Tri Reagent (MRC; TR 118) according to the manufacturer’s protocol. Complementray DNA (cDNA) was synthesized using the High Capacity cDNA Reverse Transcription Kit (Applied Biosystems; catalog no.: 4368814) with Oligo(dT)_20_ Primer (Invitrogen; catalog no.: 18418020). qPCR was run on a QuantStudio 7 Flex (Applied Biosystems) using 40 ng cDNA, Fast SYBR Green Master Mix (Applied Biosystems; catalog no.: 4385610), and 400 nM primers. Primers are listed in Table S5. Fold change was calculated using the ΔΔCt method with GAPDH or 18S as the housekeeping gene.

### Metabolomics

Steady-state levels of metabolites were measured as described before (64). Chromatographic separation was accomplished by hydrophobic interaction liquid chromatography using a Luna NH2 3 μm, 2 mm × 100 mm column, (Phenomenex) on a Vanquish Flex Quaternary UHPLC system (Thermo Fisher Scientific). For MS analyses an Orbitrap Fusion Lumos Tribrid mass spectrometer (Thermo Fisher Scientific) interfaced with an H-ESI, electrospray source (Thermo Fisher Scientific) was used. Data were collected for each sample in negative mode using two different mass ranges (70–700 and 220–900 *m/z*) to enhance sensitivity for larger less abundant compounds and in positive mode (70–900 *m/z*). The data were referenced to the MZCloud (mzcloud.org) and Mass Bank (massbank.eu) databases. All data were converted to MZXML format using MassMatrix. Peak areas, including isotopically enriched metabolites, were obtained using Mave for targeted analysis. Differential abundance of metabolites was computed in R using the student’s *t* test to determine significance and log_2_ fold change to determine magnitude and direction of change. Samples were quantile normalized using the R package *preprocessCore* before computing these metrics. FDR-corrected *p* values were also computed to adjust for multiple-testing bias.

### Human ccRCC specimens and immunocytochemistry

ccRCCs and normal kidney tissues used for measurements of Zn were deidentified specimens from never smoking Caucasian males described previously (64). For immunocytochemistry, sections of fixed and paraffin-embedded tumors were processed in the Pathology Research Core at Cincinnati Children's Hospital Medical Center and analyzed by light microscopy. LAMP1 HPA014750 antibody was used (Sigma).

### Statistical analysis

Bar plots were prepared using the R package *ggplot*. Mean and standard deviation were computed using base R functionality in conjunction with the package *dplyr*. Statistical tests for intergroup comparison utilized the independent two-tailed *t* test except for comparisons in which one set of values were limited to the value of one in which case the one-sample *t* test was utilized (mu = 1). *t* Test functionality employed utilized the base R *stats* package.

## Data availability

Data from RNA-Seq experiments are presented in Tables S1–S3. Data from the metabolomic analysis are presented in Table S4. Additional information or raw data will be shared upon request addressed to the contact author.

## Supporting information

This article contains supporting information.

## Conflict of interest

The authors declare that they do not have conflicts of interests with the content of this article.
