# Supplementary material for: Microtubule-associated protein MAP1LC3C regulates lysosomal exocytosis and induces zinc reprogramming in renal cancer cells
Source: J Biol Chem. 2023 Mar 30;299(5):104663. doi: 10.1016/j.jbc.2023.104663 (PMC10173779; doi:10.1016/j.jbc.2023.104663)
Supplement: Supporting Table S5 [file mmc5.docx]

| Supporting Table 5. | | |
| --- | --- | --- |
| Primer Sequences |  |  |
| **Gene** | **Forward Primer** | **Reverse Primer** |
| LAMP1 | TACAATTCTTCCTGACGCGAGACC | TCCGCGTTGCACTTGTAGGAATTG |
| LAMP2 | TGGCAATGATACTTGTCTCTGGC | AGCTGCCTGTGGAGTGAGTTGAT |
| CTSD | TGATTCAGGGCGAGTACA | GGACAGCTTGTAGCCTTTG |
| MCOLN1 | CCACAAGCTGGTCAATGT | TCAGGACGCTGAAGGTATAG |
| NEU1 | TCCAGAGTTCCGAGTGAA | GGTTGCCAGGGATGAATAG |
| TFEB | ATGCCCACCACGCTACC | ATCTGTGAGCTCTCGCTTC |
